# Temporal trends and future projections of cysticercosis-induced epilepsy: insights from the global burden of disease study 2021- a cross-sectional study

**DOI:** 10.3389/fpubh.2025.1576226

**Published:** 2025-05-19

**Authors:** Siyuan Yang, Xiaoyu Ji, Xuebo Sun

**Affiliations:** Department of Neurosurgery, The First Affiliated Hospital of Soochow University, Suzhou, China

**Keywords:** cysticercosis, epilepsy, global burden of disease, public health, epidemiology

## Abstract

**Background:**

The burden of disability and a significant portion of early deaths linked to Cysticercosis are primarily due to epilepsy. This research sought to clarify the temporal patterns and forecast the future prevalence and years lived with disability (YLDs) associated with Cysticercosis-induced epilepsy (CIE), providing crucial information for the development of targeted prevention and treatment strategies.

**Methods:**

Data from the 2021 Global Health database were used to measure the global, regional, and national burden of CIE by country, region, age, gender, and sociodemographic index (SDI). Age-period-cohort mode, the Auto Regressive Integrated Moving Average (ARIMA) model, and joinpoint regression analysis were also carried out.

**Results:**

The global prevalence and YLDs cases of CIE increased from 1992 to 2021, marking a 36.1 and 13.9% increase. However, the global prevalence and YLDs rates of CIE declined from 1992 to 2021, with estimated annual percentage change (EAPC) of −1.281 (95% CI: −1.373 to −1.19) and −1.878 (95% CI: −1.961 to −1.794). The age effect across global and SDI regions demonstrates an upward trend with advancing age, while unfavorable period effects are evident in high-SDI regions, where risk ratios for prevalence and YLDs exceed 1. The ARIMA model predicts a global rise in the total number of prevalence and YLDs cases from 2021 to 2036, with estimates reaching 4,955,416 (95% UI: 4,739,974 to 5,170,858) for prevalence and 2,032,208 (95% UI: 1,408,920 to 2,655,495) for YLDs by 2036.

**Conclusion:**

This study elucidates the complex epidemiological landscape of CIE, noting a global increase in prevalence and YLDs number against a decline in rates. Over the next 15 years, the burden of CIE is expected to remain significant, with high SDI regions warranting particular focus. The findings emphasize the necessity for region-specific strategies to mitigate the projected growth of CIE, highlighting the importance of tailored interventions.

## Introduction

Cysticercosis, a parasitic infection caused by the larval form of the pork tapeworm *Taenia solium* (*T. solium*), is a major public health issue, especially in regions with inadequate sanitation and limited healthcare access (1, 2). According to previous report of the Global Burden of Disease (GBD) 2021 database (3) (pp. 1990-2021), there were an estimated 4.357 million cases of cysticercosis worldwide. Pigs typically act as the intermediate hosts, and humans become infected by ingesting *T. solium* eggs, often through contaminated food or water. This leads to the formation of cysticerci in various tissues (4, 5). The condition is widespread in Latin America, Sub-Saharan Africa, and parts of Asia, particularly in rural areas where pig farming occurs in close proximity to humans under poor sanitary conditions (6). Factors such as inadequate sanitation, substandard pig farming and slaughtering practices, unsafe cooking methods, and inefficient wastewater treatment systems perpetuate the cycle of contamination (7–9).

Neurocysticercosis (NCC), the infection of the central nervous system by *T. solium* larvae, represents the most severe form of cysticercosis and is a leading cause of epilepsy in endemic regions (10, 11). Epilepsy attributable to NCC accounts for one-third of all epilepsy cases within these populations (9). However, accurately determining the prevalence of NCC-induced epilepsy is challenging due to the diverse clinical presentations and the limited availability of diagnostic resources in many high-burden areas (12).

Cysticercosis-induced epilepsy (CIE) is triggered by inflammation in the brain parenchyma caused by the degeneration of cerebral cysticerci (10, 13). Epilepsy in NCC patients is most commonly associated with degenerated cysts, though living and calcified cysts are also involved (14, 15). Additionally, the release of antigens and toxins during cyst degeneration, along with local inflammation, brain compression, and scar formation, significantly increases the risk of seizures. Residual calcifications, perilesional gliosis, and antigenic exposure further contribute to seizure recurrence (11, 16–21). Recurrent seizures may result in hippocampal sclerosis, which is linked to mesial temporal lobe epilepsy (22–24). The damage caused by epilepsy goes beyond its direct impact on the brain and can significantly lower a patient’s quality of life. Frequent seizures may affect cognitive function, emotional well-being, and daily activities, increasing the sense of social isolation (13).

Epidemiological studies has quantified this substantial burden. A meta-analysis revealed a pooled prevalence of 17% (95% CI: 14–20%) for porcine cysticercosis across the included studies (25). Neurocysticercosis and epilepsy imposed significant disease burdens (26, 27), with mean disability-adjusted life years (DALYs) of 3.0 and 3.9 per 1,000 person-years, respectively (28). In Mexico, 144,433 individuals are estimated to suffer from NCC-associated epilepsy (29). This parasitic infection remains a major etiological factor for acquired epilepsy in tropical endemic regions, accounting for approximately 30% of late-onset seizure disorders in these areas (30).

However, there is few studies comprehensively analyzing the prevalence and years lived with disability (YLDs) associated with CIE based on the GBD database, leaving a gap in understanding the broader impact of the condition on public health. Further research is urgently needed to quantify the burden of CIE and to inform effective prevention and management strategies.

## Methods

### Data source and disease definition

All data are available in Global Health Data Exchange,^1^ which is a comprehensive database that evaluates the global incidence, prevalence, YLDs, DALYs and healthy life expectancy (HALE) for 371 diseases and injuries across 204 countries and territories conducted by the Institute for Health Metrics and Evaluation (IHME) (31).

In GBD 2021, epilepsy was categorized as idiopathic (unknown or genetic cause) and secondary (known cause, such as brain structural or chemical abnormalities). Secondary epilepsy burden was calculated through a multi-step modeling approach. First, data from systematic reviews, population-based surveys and clinical studies were used to estimate epilepsy proportions, with adjustments made for diagnostic method variations (e.g., magnetic resonance imaging(MRI) or computerized tomography(CT) usage). Then, a mixed-effects model combined with Disease Modeling Meta-Regression (DisMod-MR) 2.1 was then employed to disaggregate secondary epilepsy burden from total epilepsy estimates, further allocating it to specific causes (e.g., meningitis, cysticercosis) (32). Further details on the methodology of epilepsy could be seen elsewhere (33) or in the website.^2^

Cysticercosis, caused by *T. solium* from contaminated food or water, leads to larval cysts in the central nervous system that may cause epilepsy, diagnosed by MRI or CT. The International Classification of Diseases, Tenth Revision (ICD-10) codes for cysticercosis are B69-B69.9. The non-fatal estimation for cysticercosis focused on estimating prevalence of NCC among epileptics at risk as well as the prevalence of NCC with epilepsy which estimated by combining data from a systematic literature review with modeling techniques. In detail, studies reporting the prevalence of NCC among people with epilepsy were identified, and meta-regression-Bayesian, regularized, trimmed (MR-BRT) was used to adjust for differences in diagnostic definitions. DisMod-MR then modeled NCC prevalence among at-risk epileptics, incorporating factors like pig farming, socio-demographic index (SDI), and Muslim population proportion. Adjustments for sanitation access and religious practices refined the at-risk population, and the fraction of epilepsy due to NCC was estimated by combining overall epilepsy prevalence with NCC prevalence in this group. YLDs were then calculated as prevalence multiplied by the category-specific disability weight. Further details have been published in the website online.^3^

The Retrieval strategy of data analyzed in the study is as follows:“GBD estimate”:impairment;“Measure”: Prevalence, YLDs; “Metric”: number, percentage, rate; “Impairment”: epilepsy; “Cause”: Cysticercosis; “Location”: global, 5 SDI regions, 21 regions and 204 countries; “Age”: all ages, age-standardized, <5 years to >95 years; “Sex”: both, female, male; “Year”: from 1992 to 2021.

### Concept definition

The SDI value, introduced by the IHME in 2015, serves as a composite measure to evaluate the development levels of countries or regions, highlighting the link between societal progress and population health outcomes. In the GBD 2021 analysis, 204 countries and territories are classified into five SDI categories: low (0 to 0.2), low-middle (0.2 to 0.4), middle (0.4 to 0.6), high-middle (0.6 to 0.8), and high (0.8 to 1.0) (27).

YLDs is a metric used to measure the impact of non-fatal health issues by calculating the number of years individuals live with disabilities due to diseases or injuries. It is measured by taking the prevalence of the condition multiplied by the disability weight, which reflect severity of different conditions on a scale from 0 (perfect health) to 1 (death). Disability weights are developed through derived from multinational surveys and refined using probit regression, enabling quantification of both the frequency and impact of health conditions (32, 34). The age-standardized rate (ASR) adjusts for differences in age distribution, enabling fair comparisons of health outcomes across different populations and time periods. The age-standardized prevalence rate (ASPR) reflects the number of cases per 100,000 individuals, adjusted for age, while the YLDs per 100,000 population is represented by the age-standardized YLDs rate (ASYR). Uncertainty is propagated through each computation step by sampling 500 draws at each step. By ordering the draws, the 95% uncertainty intervals (UIs) were derived from the 25th and 975th percentiles of the ordered 500 estimates, adhering to the GBD algorithm (31, 33).

The estimated annual percentage change (EAPC) is a widely recognized and effective metric for analyzing trends in parameters, prevalence and YLDs over defined time periods (19, 20). The 95% confidence intervals (CIs) for the EAPC are derived from this fitted model. A trend is increasing if the 95% CIs lower limit is above 0, decreasing if the upper limit is below 0, and not significant if the CIs includes 0. This study also employs percentage change to reflect the variations in prevalence, and YLDs cases in 2021 compared to 1992 (percentage change = (2021 cases−1992 cases) / 1992 cases) (35, 36).

### Statistical analysis

We used statistical methods to analyze CIE burden. Correlation analysis is employed to evaluate the linear relationship between ASR and SDI by determining the correlation coefficient. The age-period-cohort model was applied to investigate trends in CIE by examining three primary factors: age, period, and cohort. Age effects capture changes in disease occurrence due to aging, period effects reflect influences such as advancements in diagnosis or treatment that affect all age groups within a specific time frame, and cohort effects identify variations in disease rates across different birth cohorts. To mitigate collinearity among these factors, a sum-to-zero constraint was used, enabling the estimation of both net drift (overall long-term trends) and local drift (short-term deviations). The analysis stratified age, period, and cohort into 5-year intervals (37–39).

To predict disease progression over the next 15 years, focusing on prevalence, and YLDs rates from 2022 to 2036, We applied the Auto Regressive Integrated Moving Average (ARIMA) model through R’s “forecast 8.22.0”^4^ and “tseries 0.10.55”^5^ packages to perform our analysis (40, 41). The ARIMA (p, d, q) model was used to analyze time series data (42)(pp. 2006–2016), ensuring stationarity via differencing and assessing it with autocorrelation function (ACF) and partial autocorrelation function (PACF) plots. The optimal model was selected using auto.arima() based on Akaike information criterion (AIC). Residual normality was checked, while the Ljung–Box test assessed serial correlation.

Joinpoint regression analysis with the Joinpoint Regression Program, version 4.9.1.0 (National Cancer Institute, Rockville, MD, United States) was used for analyzing trends. This approach defines epidemiological trends by computing the annual percentage change (APC), the average annual percentage change (AAPC), and their respective 95% CIs (43, 44). In the Joinpoint software, the Final Selected Model which refers to the best-fitting model chosen based on statistical criteria, such as minimizing the Bayesian Information Criterion (BIC), was selected. A statistically significant *p*-value was less than 0.05.

Data preparation, analysis, and graph generation were conducted with R software (version 4.3.1). The ggplot2 package was used to create visualizations, while final refinements were made with Adobe Illustrator (version CS5). Statistical significance was denoted by a two-tailed *p*-value of less than 0.05.

## Results

### Global trends

**Figure 1:**
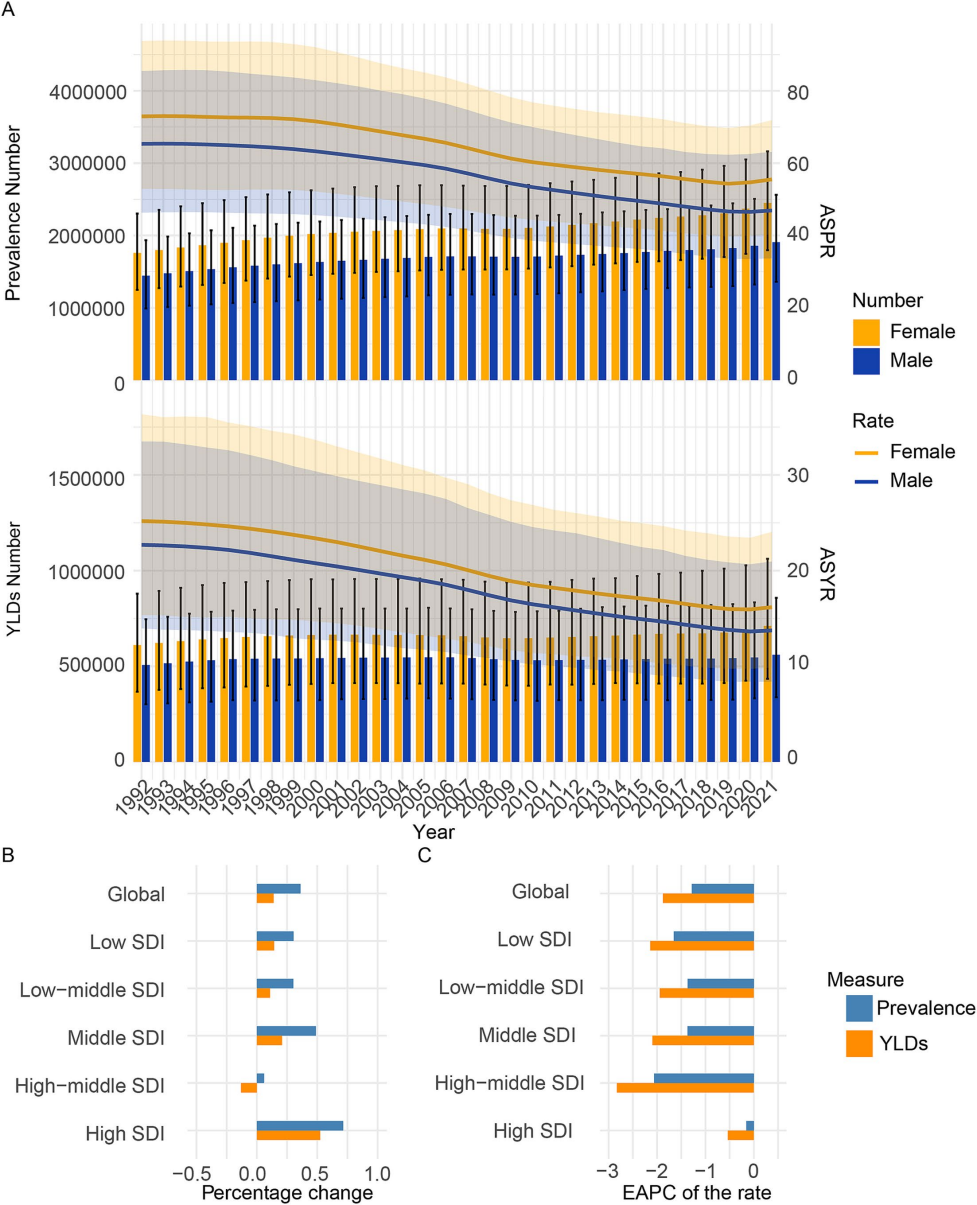
Temporal trend of CIE burden in global and 5 SDI regions. **(A)** Prevalence, and YLDs cases and rates from 1992 to 2021. **(B)** Percentage change in cases of prevalence, and YLDs in 1992 and 2021. **(C)** The EAPC of prevalence and YLDs rates from 1992 to 2021. SDI, Socio-demographic index; EAPC, Estimated Annual Percentage Change; YLDs, Years lived with disability.

The global prevalence cases of CIE increased from 3200.1 thousand cases (95% UI: 2225.4 thousand to 4200.9 thousand cases) in 1992 to 4357.2 thousand cases (95% UI: 3150.3 thousand to 5716.4 thousand cases) in 2021, marking a 36.1% increase. However, the global ASPR of CIE fell from 68.9 (95% UI: 49.2 to 89.5) per 100,000 people in 1992 to 51.3(95% UI: 37.2 to 67.3) per 100,000 people in 2021, with an EAPC of −1.281 (95% CI, −1.373 to −1.19) (Figure 1, Supplementary Table S1).

The global YLDs cases of CIE increased from 1116.2 thousand cases (95% UI: 666.3 thousand to 1621.8 thousand cases) in 1992 to 1271.1 thousand cases (95% UI: 773.5 thousand to 1919.4 thousand cases) in 2021, marking a 13.9% increase. Additionally, the global ASYR of CIE fell from 23.8 (95% UI: 14.7 to 34.7) per 100,000 people in 1992 to 15 (95% UI: 9.1 to 22.5) per 100,000 people in 2021, with an EAPC of −1.878 (95% CI: −1.961 to −1.794) (Figure 1, Supplementary Table S2).

### SDI regional trends

Compared to 1992, the prevalence number in 2021 increased across all five SDI regions. In 1992–2021, the middle SDI regions reported the highest absolute numbers of prevalence cases of CIE and the low SDI regions reported the least absolute numbers of prevalence. Over the three decades from 1992 to 2021, the burden of CIE has shown varied patterns across these regions. The low, low-middle, middle, high-middle SDI regions have seen a steady rise in prevalence number, while the high SDI region has experienced a more fluctuating trend, with an initial increase followed by a subsequent decrease in prevalence. In the early 1990s, the high SDI group had the lowest ASPR. A slight increase occurred from 1990 to 2000, followed by a gradual decline after 2000, likely due to better disease management and healthcare. Despite the decline, high SDI remains the category with the lowest ASPR, highlighting superior healthcare infrastructure and early disease detection (Figure 1, Supplementary Figure S1). Furthermore, in 1992–2021, the low SDI regions reported the highest prevalence rates of CIE and the high SDI regions reported the least prevalence rates (Figure 1, Supplementary Figure S1).

Compared to 1992, the YLDs number in 2021 increased across all five SDI regions except the high-middle SDI regions. In 1992–2021, the middle SDI regions reported the highest absolute numbers of YLDs cases of CIE and the high SDI regions reported the least absolute numbers of YLDs. Over the three decades from 1992 to 2021, the burden of CIE has shown varied patterns across these regions. The low, low-middle, middle, high SDI regions have seen a overall rise in YLDs number, while the high-middle SDI region has experienced a more fluctuating trend, with an initial increase followed by a subsequent decrease in YLDs (Figure 1, Supplementary Figure S1). Furthermore, in 1992–2021, the low SDI regions reported the highest YLDs rates of CIE and the high SDI regions reported the least YLDs rates (Figure 1, Supplementary Figure S1).

### GBD regional trends

**Figure 2:**
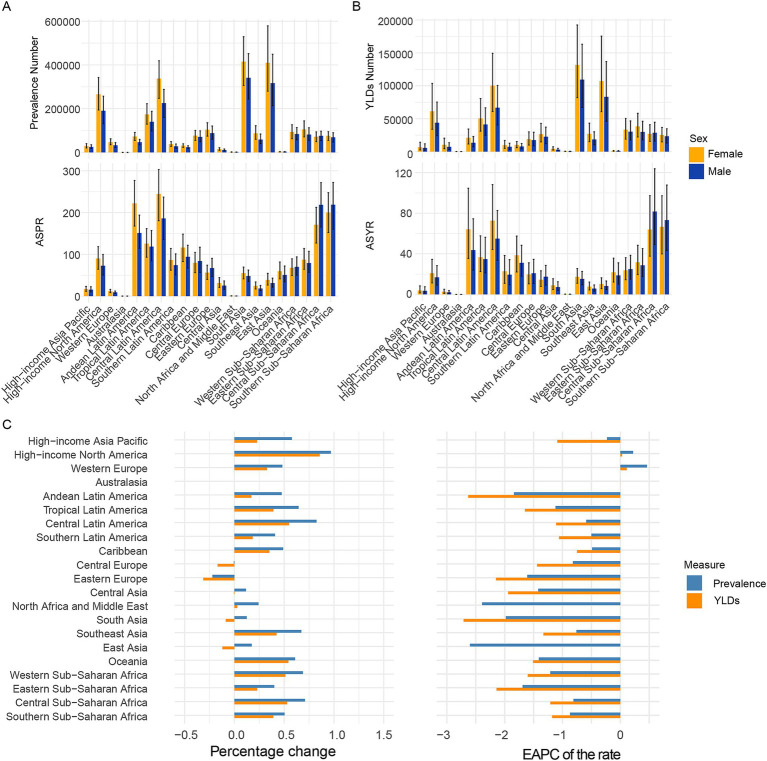
Temporal trend of CIE burden in 21 regions. **(A)** Prevalence cases and rates in 2021. **(B)** YLDs cases and rates in 2021. **(C)** Percentage change in cases of prevalence and YLDs in 1992 and 2021, and the EAPC of prevalence and YLDs rates from 1992 to 2021. SDI, Socio-demographic index; EAPC, Estimated Annual Percentage Change; YLDs, Years lived with disability.

Figure 2 and Supplementary Tables S1, S2 showed the prevalence and YLDs number and ASR in 21 GBD regions. In 2021, the highest three regions in prevalence and YLDs number were South Asia, East Asia, Central Latin America which may because of the three populous countries (India, China, United States of America). The lowest three regions in prevalence and YLDs number were Australasia, Oceania, North Africa and Middle East which may because of geographical location or religious dietary habits. The highest five regions in ASPR and ASYR is Southern Sub-Saharan Africa, Central Sub-Saharan Africa, Central Latin America, Tropical Latin America, Andean Latin America. The lowest five regions in ASPR and ASYR is Australasia, North Africa and Middle East, Western Europe, High-income Asia Pacific, Southeast Asia.

Over the past 30 years, increases in prevalence numbers have been observed in more than half of the regions except Australasia, Central Europe, Eastern Europe and increases in YLDs numbers have also been observed in more than half of the regions except Australasia, Central Europe, Eastern Europe, Central Asia, South Asia, East Asia. Increases in ASPR and ASYR have been observed in more than half of the regions except Australasia, Western Europe and High-income North America.

As to sex pattern, the number and rate in female is more than that in male in all age patterns except the 95 + age group. Furthermore, the number and rate in female is more than that in male in most of the 21 regions.

### National trends

**Figure 3:**
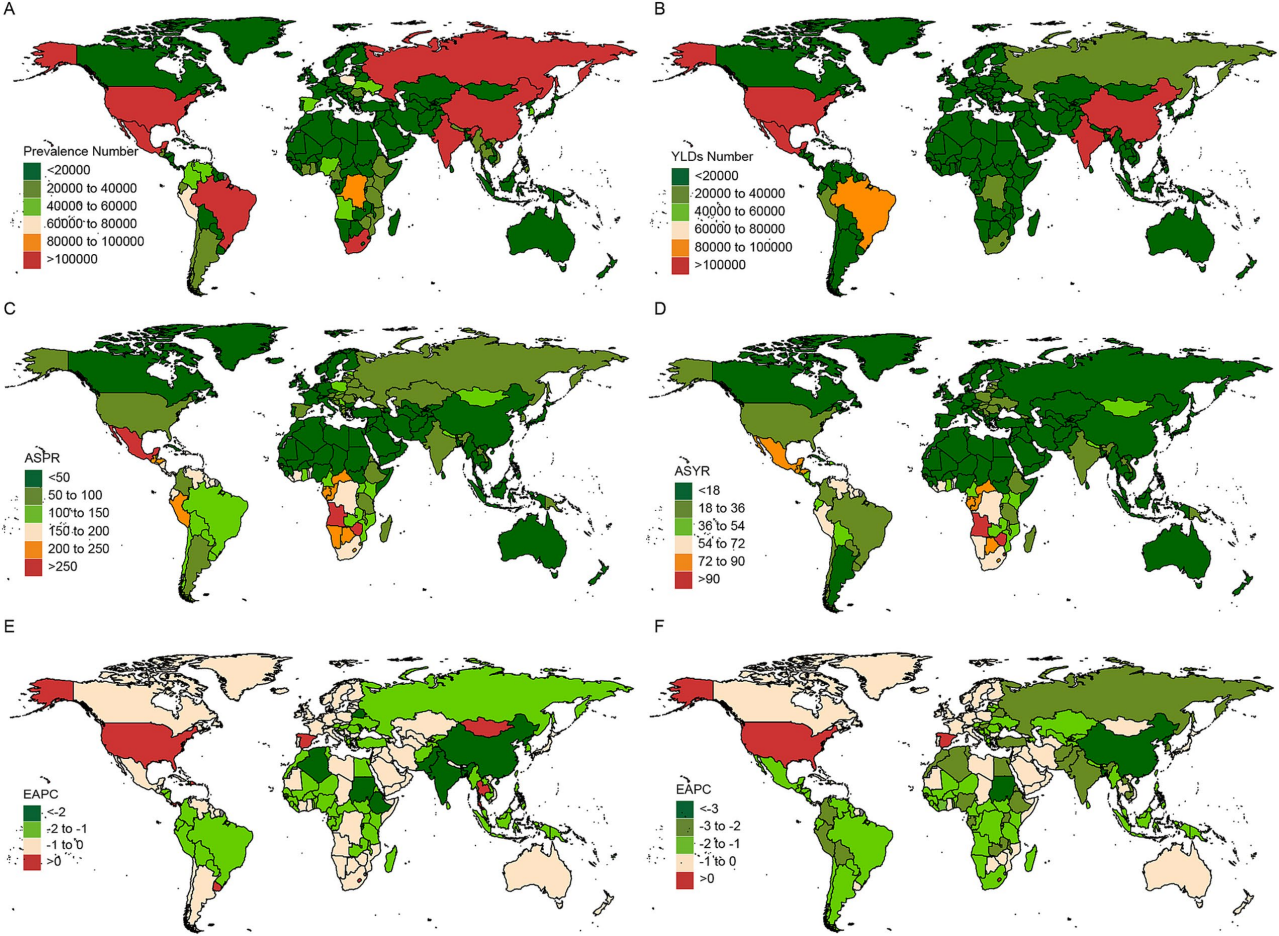
Temporal trend of CIE fractures burden in countries level. Number in prevalence **(A)**, and YLDs **(B)** cases across 204 countries in 2021. ASR in prevalence **(C)**, and YLDs **(D)** across 204 countries in 2021. EAPC in prevalence **(E)**, and YLDs **(F)** rates across 204 countries from 1992 to 2021. EAPC, Estimated Annual Percentage Change; YLDs, Years lived with disability.

Figure 3 and Supplementary Tables S3, S4 display the prevalence, YLDs, ASR, and EAPC for CIE across 204 countries over the past three decades. In 2021, seven countries reported prevalence cases exceeding 100,000, accounting for 63.77% of the global prevalence, with a marked geographic concentration across almost all five SDI regions. Notably, India, China and United States of America, as the demographic titans, claimed the top three rankings in terms of prevalence numbers. Half of the countries and territories were found to have ASPRs exceeding the global average, with Mexico, Cabo Verde, Angola, Zimbabwe, and Eswatini ranking as the top five. A detailed analysis of the SDI stratification, as shown in Supplementary Figure S5, reveals a more complex and diverse pattern of CIE prevalence across different nations.

The EAPC analysis of prevalence rates revealed that, among the 204 countries and territories, eight countries - Dominican Republic, Israel, Lesotho, Panama, Spain, Thailand, United States, and Uruguay - showed an upward trend in CIE prevalence. Notably, most of these countries are situated in high and upper-middle SDI regions, which are predominantly marked by advanced industrial development. Among the nations with the fastest increasing prevalence, Lesotho is at the forefront with an EAPC of 0.776 (95% CI: 0.572 to 0.98), closely followed by Spain and Uruguay. Conversely, the Democratic People’s Republic of Korea, Sudan, and China ranked highest among the countries with declining prevalence rates.

In 2021, out of the 204 countries and territories, four reported over 100,000 years lived with disability (YLDs) cases, collectively accounting for 49.4% of the global YLDs with a pronounced geographic concentration. Notably, India, China and United States of America were also claimed as the top three rankings in terms of YLDs numbers. Over half of the countries and territories were found to have ASYRs exceeding the global average, with Zimbabwe, Angola, Eswatini, the Central African Republic, and Lesotho ranking as the top five (Supplementary Tables S3, S4, Supplementary Figure S5).

The EAPC analysis of YLD rates revealed that China led the declining trend, with an EAPC of −3.688 (−3.955 to −3.42), marking the most significant reduction among the 204 countries and territories. In contrast, three countries - Lesotho, Spain, and the United States - demonstrated an upward trend in CIE YLDs over the same period.

### Sex-specific burden of CIE

As to sex-specific burden, a higher burden of CIE was seen in females than in males globally (Figures 1, 2, Supplementary Figure S2–S4), which might be due to physiological and immune differences, higher exposure risk, neurophysiological factors, and potential reporting biases. As to 21 GBD regional, most of 21 regions presents a higher burden of CIE in females than in males.

### The association between CIE burden and SDI

**Figure 4:**
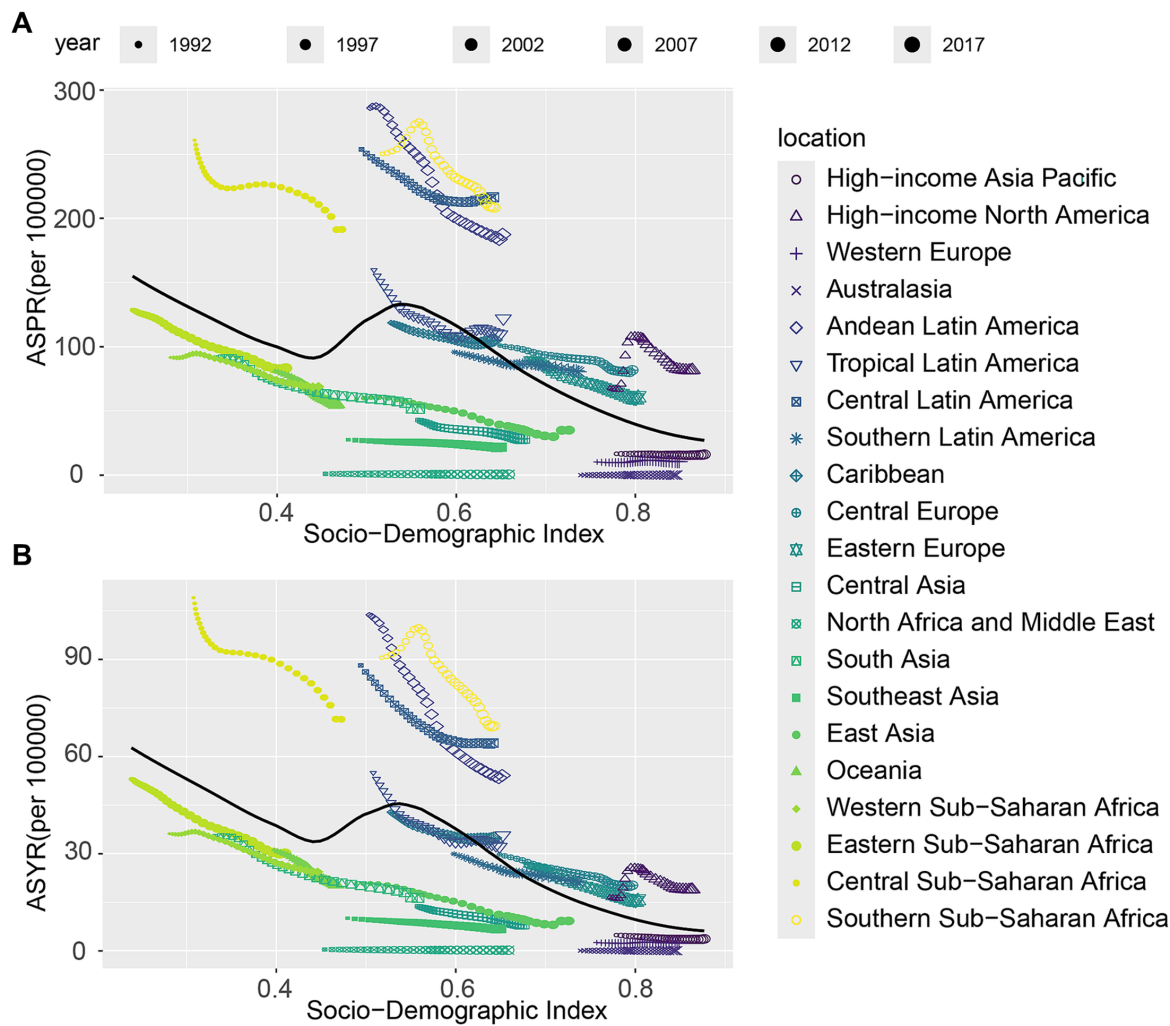
The associations between the SDI and ASR per 100,000 population of CIE across 21 GBD regions. **(A)** Prevalence; **(B)** YLDs. SDI, Socio-Demographic Index; GBD, Global Burden of Disease; YLDs, Years lived with disability.

In this study, a scatter plot with trend lines was used to explore the correlation between CIE and the SDI across 21 regions (Figure 4) and 204 countries (Supplementary Figure S5). The results reveal that CIE prevalence rates, and YLDs rates decrease followed by the rise of the SDI value. Regions such as Southern Sub-Saharan Africa, Central Sub-Saharan Africa, Central Latin America, Tropical Latin America, Andean Latin America have a higher burden than expected. However, Australasia, North Africa and Middle East have a lower burden than expected. In 204 countries, zimbabwe, Angola, Eswatini, Central African Republic, Lesotho have a higher burden than expected.

### Age, period and birth cohort effects on CIE disorders prevalence and YLDs

**Figure 5:**
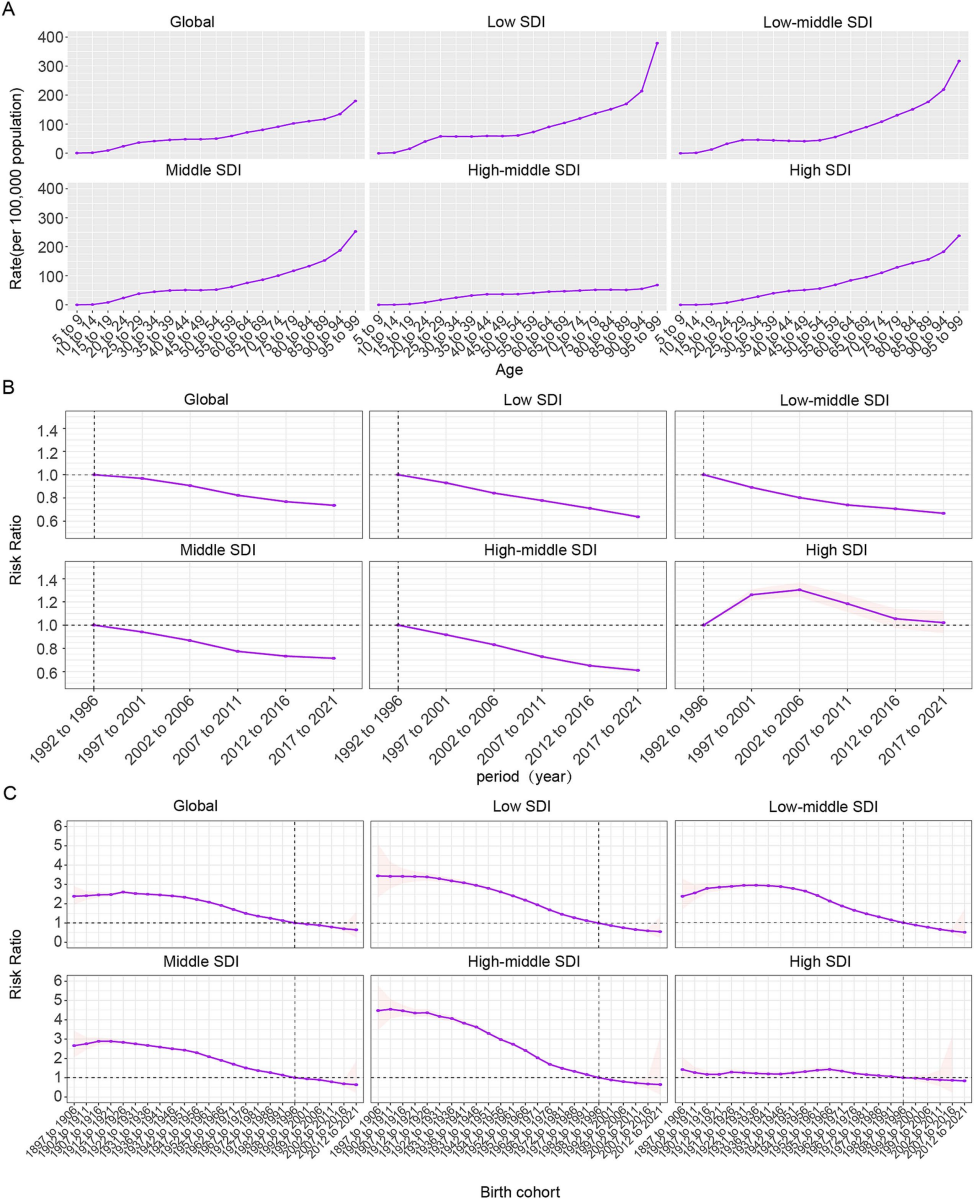
Age-period-cohort effects on CIE prevalence globally. **(A)** Age effects are shown by the fitted longitudinal age curves of prevalence (per 100,000 person-years) adjusted for period deviations. **(B)** Period effects are shown by the relative risk of prevalence (prevalence rate ratio) and computed as the ratio of age-specific rates from 1992 to 1996 (the referent period) to 2017–2021. **(C)** Cohort effects are shown by the relative risk of prevalence and computed as the ratio of age-specific rates from the 1895 cohort to the 2012 cohort.

Age, period, and birth cohort effects on the prevalence and YLDs of CIE, as derived from the age-period-cohort model, are shown in Figure 5 and Supplementary Figure S6. The age effect across SDI regions exhibited a consistent trend, with the greatest risk noted among individuals aged 95 to 99, alongside a steady rise in risk as age progressed. Notably, the high-middle SDI region displayed lower prevalence and YLD rates across all age categories in comparison to other SDI regions, with relatively small disparities between age groups within this region (Figure 5A, Supplementary Figure S6A).

Overall, the period effect revealed a steady decline in both ASPR and ASYR worldwide, a trend mirrored in the five SDI regions, with the exception of high-SDI areas. During the study period, high-SDI regions predominantly faced unfavorable period risks, while regions with lower SDI levels generally demonstrated more favorable period risks. When compared to those in the reference period of 1992–1996, the relative period risk for individuals during the 1997–2001, 2002–2006, and 2007–2011 periods for prevalence was 1.261 (95% CI: 1.216 to 1.308), 1.304 (95% CI: 1.244 to 1.366), and 1.184 (95% CI: 1.113 to 1.259) and for YLDs were 1.194 (95% CI: 1.151 to 1.238), 1.204 (95% CI: 1.149 to 1.262), and 1.074(95% CI: 1.01 to 1.142) were in high SDI region (Figure 5B).

The birth cohort effect revealed a global pattern of initially rising prevalence and YLDs risk across consecutive cohorts, followed by a decline, indicating an overall downward trend. This trend was particularly evident in middle SDI regions, with continuous improvement observed across five SDI regions. For cohorts born before 1942–1951, reductions in risk were minimal, but a significant decline was seen afterward. Individuals born before 1987–1996 faced higher risks compared to this cohort, while those born later experienced relatively lower risks.

### Global disease burden prediction for CIE to 2036

**Figure 6:**
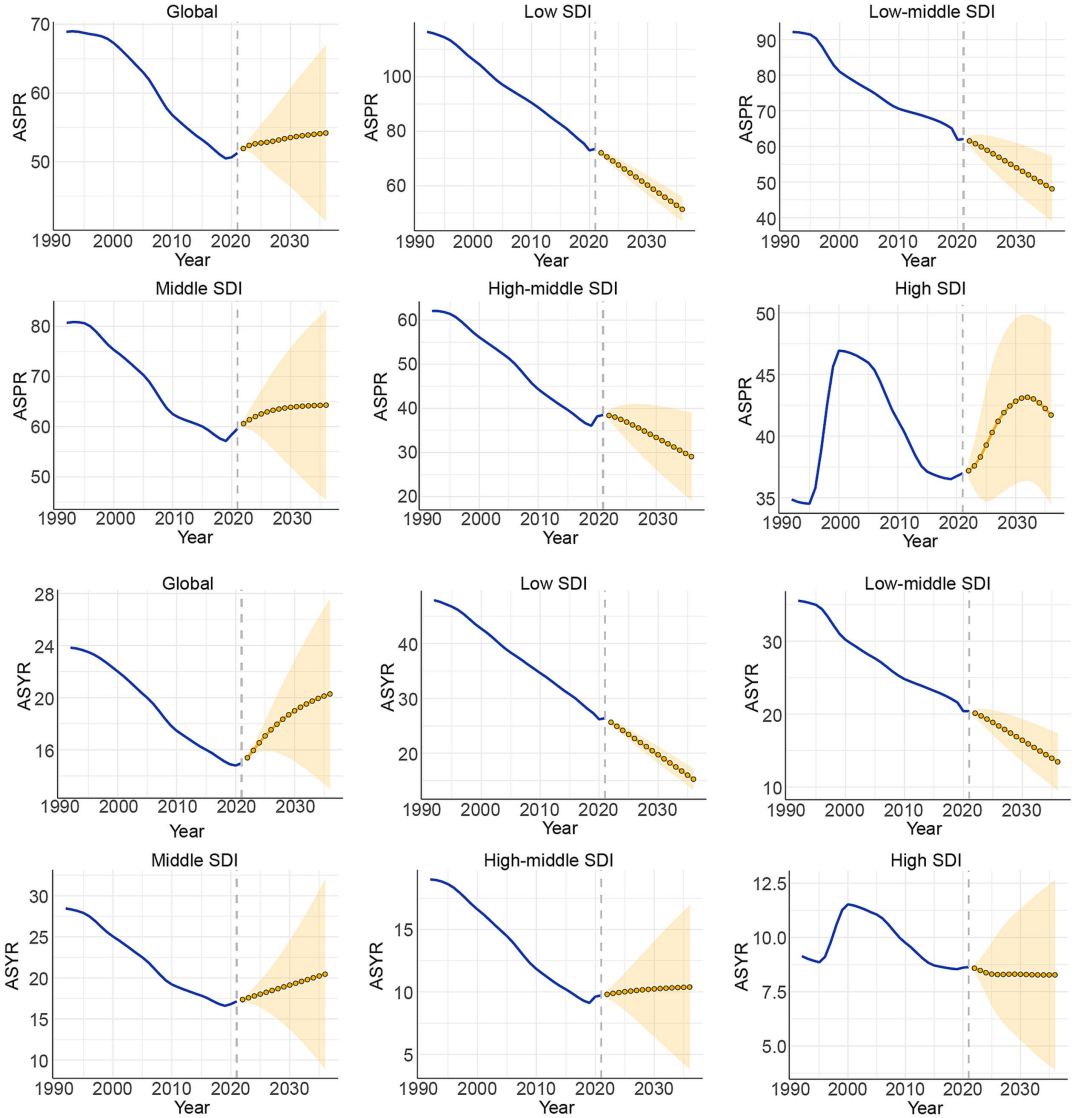
ASPR and ASYR of CIE in global and 5 SDI region from 1992 to 2036.

The ARIMA model was utilized to predict future trends in ASR and the number of cases related to CIE from 2022 to 2036 (Figure 6, Supplementary Figure S7). The model forecasts a global rise in the total number of prevalence and YLDs cases between 2021 and 2036, with estimates reaching 4,955,416 cases (95% UI: 4,739,974 to 5,170,858) for prevalence and 2,032,208 cases (95% UI: 1,408,920 to 2,655,495) for YLDs by 2036. Meanwhile, the ASPR and ASYR are anticipated to show a gradual upward trend, reaching 54.19 (95% UI: 41.43 to 66.95) and 20.28 (95% UI: 13.01 to 27.56), respectively. However, these rates will stay below the historical peak ASR. Additionally, a projected rise in the total number of prevalence and YLDs cases is expected in low, low-middle, and middle SDI regions between 2021 and 2036. However, it is stable in high and high-middle SDI regions. A projected increase in the ASPR and ASYR were also shown in low, low-middle SDI regions from 2021 to 2036.

### Joinpoint regression analysis

We employed the joinpoint regression model to detect shifts in the phase trends of the global burden of CIE. Supplementary Figure S8, Supplementary Tables S6, S7 presents the trends in prevalence, and YLDs related to CIE over the past three decades in global and the five SDI regions. Overall, the ASPR exhibited a general downward trend globally and across different SDI regions except the high SDI regions and the ASYR exhibited a general downward trend globally and across all the five SDI regions according to the AAPC values from 1992 to 2021. During the COVID-19 pandemic (2020–2021), ASPR and ASYR rose in high-middle and middle SDI regions but declined in low-middle and low SDI regions. Globally, prevalence numbers increased across all SDI levels, while ASYR generally declined except in high-middle SDI regions. Prevalence and YLDs rose in high-middle and middle SDI areas but decreased in low-middle SDI regions.

## Discussion

The study revealed a steady annual increase in the global prevalence and YLDs number of CIE from 1992 to 2021, despite a decline in ASR. In the study, lower SDI regions exhibited higher rates of CIE in comparison to higher SDI regions. The age-related increase in CIE risk was consistent globally and across all SDI regions, with risk escalating as individuals aged. However, the period effects in high-SDI regions diverged from those observed in other SDI regions, showing less favorable trends. There was considerable variation in the changes in CIE prevalence and YLDs between countries. Predictive models forecast a continued rise in both the total number of cases and age-standardized rates of CIE prevalence and YLDs over the next 15 years, underscoring the need for region-specific prevention and control strategies.

CIE poses a significant challenge to global public health (45). Globally, neurocysticercosis is a significant contributor to acquired epilepsy (46). In countries where it is endemic and population-based studies are available, neurocysticercosis is responsible for almost one-third of all epilepsy cases (47–49). The burden of disability and a significant portion of early deaths linked to *T. solium* infection are primarily due to epilepsy (50). Understanding the burden and trends of *T. solium* cysticercosis is crucial for disease assessment and improving clinical outcomes of CIE. *T. solium* cysticercosis, caused by ingesting eggs through contaminated food or water, remains endemic in regions like sub-Saharan Africa, Latin America, South Asia, and Southeast Asia (8). Poor sanitation, inadequate hygiene, free-roaming pigs, lack of meat inspection, and the consumption of undercooked pork elevate infection risk (45). Global prevalence and YLDs have increased due to demographic shifts, while a decline in ASR reflects the success of control measures. Improved sanitation, infrastructure, and antiparasitic treatments in rural areas have contributed to reduced prevalence across all SDI regions (10). However, an increase in prevalence rate has been observed in two high-SDI regions, namely High-income North America and Western Europe, among 21 regions. This rise is likely associated with factors such as travel and immigration (51, 52).

As to the mechanisms underlying epilepsy of NCC, we have further discussed and elaborated in detail. As reported, Cysts at any developmental stage with degenerated cysts being the most common, the release of large amounts of antigens and toxins during cysticercus degeneration, local inflammation around the cyst or calcification, and the formation of scars, as well as reactive gliosis in the vicinity of calcified lesions (4, 6, 11, 15). Furthermore, some research indicates that recurrent seizures may result in hippocampal sclerosis which lead to the subsequent development of temporal lobe epilepsy (22–24). Taenia larval acetylcholinesterases can also interfere with cholinergic signaling and play a role in the NCC pathogenesis (53). As research on the underlying mechanisms of CIE deepens, progress in the diagnosis and treatment of neurocysticercosis, such as combined treatment/vaccination with oxfendazole and TSOL18, has led to a reduction in burden in some regions. However, due to economic and socio-cultural factors, some advanced diagnostic and therapeutic measures have not been implemented in many endemic countries. Addressing this gap is essential for alleviating the regional burden of CIE.

A clear trend shows rising CIE cases in low and high SDI regions, while high-middle SDI areas see a decline. Though global ASR has dropped, rates remain above average in less developed regions, underscoring the need for enhanced control. Some industrialized areas show stagnation or slight increases, possibly due to policy stabilization at low prevalence levels. These patterns, influenced by diverse economic, cultural, and policy contexts, call for context-specific interventions. NCC remains a key public health concern and a major epilepsy cause in developing countries (54). In these regions, poor sanitation increases pigs’ exposure to human feces, promoting parasite transmission. Epilepsy, a common NCC symptom, often faces strong social stigma—especially in SSA, where it is linked to beliefs in witchcraft, possession, or heredity—leading to widespread prejudice (54–57). It’s also reported that *T. solium* transmission is prevalence, particularly in meat-eating, impoverished communities, with a treatment gap exceeding 90% (58). Poor sanitation in slums shows a significant correlation with CIE prevalence (58, 59). These findings indicate that biological and socio-environmental factors contribute to CIE patterns. To address this, regions should implement educational programs to raise awareness and dispel misconceptions about NCC and CIE. Simultaneously, interventions like improved sanitation, regulated pig farming, and enhanced pork inspection are essential to interrupt transmission and support the WHO 2030 goal.

This study also researches the impact of age, period, and birth cohort on the prevalence, YLDs number and ASR trends of CIE. A uniform age effect was noted across SDI regions, showing a continuous rise in risk as age advanced. Historically, the lack of awareness, insufficient policies, and inadequate healthcare systems, along with poor living and dietary habits, contributed to the highest CIE burden in the older adult (45, 46). Over the past 30 years, a favorable period effect has been noted globally, with the risk of CIE consistently declining due to economic development and the implementation of prevention and control policies. However, high SDI regions exhibit a contrasting, unfavorable period effect, unlike other regions. In these areas, the relative risk of CIE initially increased before declining, reflecting the stagnation in disease burden progress. This adverse effect in high SDI regions may be linked to attributed to global migration patterns and changing epidemiological landscapes (60). A favorable cohort effect shows a consistent decline in CIE risk globally over the past 30 years, driven by economic development and prevention policies. However, NCC prevalence and disease burden are rising in developed countries due to migration and international travel (4). Most Western European countries have reported imported cases of human cysticercosis (61, 62), and recent studies have identified locally NCC cases, with Eastern Europe facing the highest risk (63, 64). Furthermore, the diagnostic profile of NCC in high-SDI regions has also shifted, from older, rural, non-traveling European residents to younger immigrants from endemic areas and urban-dwelling European citizens (62). In high-SDI regions, preventive measures should prioritize public health surveillance and epidemiological investigations, focusing on immigrants and high-risk groups like domestic workers from Latin America. Public awareness, education, and social worker interventions are crucial to addressing NCC risks, with emphasis on migration and cross-border transmission dynamics.

A projected increase in the total number of prevalence and YLDs cases worldwide from 2021 to 2036, reaching an estimated 4,955,416 (95% UI: 4,739,974 to 5,170,858) and 2,032,208 (95% UI: 1,408,920 to 2,655,495) by 2036. The prevalence, YLDs number and ASR are also expected to increase. These alarming trends place greater demands on healthcare infrastructure and the formulation of public health policies. As such, continuous monitoring of CIE is essential, particularly by addressing its root cause - cysticercosis. Preventive measures targeting the transmission pathways of cysticercosis, such as improving sanitation, enhancing food safety, and educating at-risk populations, are critical for controlling the disease (65). As to epileptic patients infected by cysticercosis, a tiered approach is recommended that integrates secondary prevention strategies (10). Patients should receive antiepileptic drugs to control seizures and anthelmintic treatment to target the parasitic infection, along with corticosteroids to reduce inflammation associated with intracranial lesions (10, 66). Regular follow-up imaging studies are crucial to monitor the progression of cerebral lesions and adjust treatment protocols accordingly. This multifaceted strategy ensures comprehensive management of the neurological complications arising from cysticercosis, aiming to improve patient outcomes and prevent disease escalation (13, 67). Furthermore, the study also notice the temporal change of prevalence and YLDs during the COVID-19 pandemic. The rise trends happens properly because of the routine chest X-ray investigation in some regions during the COVID-19 pandemic (57). In contrast to low-middle and low SDI regions, high-middle and middle SDI regions exhibited an increase in ASPR and ASYR during the pandemic. This trend could be attributed to underreporting and reduced healthcare accessibility, rather than an actual decline in disease burden. Many low-SDI countries experienced severe disruptions in disease surveillance and healthcare infrastructure, which may have led to an artificial drop in reported cases rather than a true epidemiological shift. Notably, high-middle SDI regions show a sustained ASYR increase, which may indicate persistent healthcare challenges in epilepsy management exacerbated by the pandemic. Furthermore, some areas face the fall trends which might also be affected by the COVID-19 pandemic including disruptions to healthcare services and reduction of finance (68). However, research in this area remains limited. Therefore, further investigation into the potential causes of the rise and fall in CIE prevalence and YLDs during the COVID-19 pandemic is crucial. Such research would be significant for enhancing the detection of hidden cases and informing emergency management strategies for CIE during future pandemics.

This study is the first to comprehensively analyze global and regional CIE prevalence and YLDs using the APC model, offering valuable insights for public health policy. However, several limitations exist. First, underdeveloped healthcare systems in low-SDI countries hinder effective diagnosis, likely leading to ASR underreporting. Underreporting NCC-related epilepsy in low-SDI settings were due to limited diagnostic capacity, incomplete case reporting, and gaps in healthcare infrastructure. While the GBD framework applies statistical modeling techniques, to adjust for missing data, inherent uncertainties remain. We emphasize the need for enhanced epidemiological surveillance and improved data collection in low-SDI regions to generate more accurate disease burden estimates in future research. Second, reliance on GBD modeling due to limited raw data introduces uncertainties in estimating age, period, and cohort effects. Third, Bayesian models are used for countries lacking data, but including new studies in future GBD updates could improve accuracy and potentially revise earlier projections. Fourth, the analysis is limited to national-level data, missing regional variations within countries. Future research should incorporate additional data sources and explore region-specific factors to enhance understanding of CIE. lastly, the limitations of the ARIMA model include its reliance on strict linearity and stationarity assumptions, along with subjective parameter selection, which may lead to overfitting or underfitting in long-term predictions (e.g., 2036). Additionally, it cannot capture nonlinear relationships or external shocks, and prediction errors accumulate over time, reducing reliability. These limitations can be mitigated through cross-validation, hybrid modeling (e.g., with machine learning), and explicit uncertainty quantification.

## Conclusion

This study identified favorable temporal trends in global CIE epidemiology, but the rising prevalence and YLDs, particularly in low-SDI regions, remain concerning. An unfavorable period effect in high-SDI regions, linked to migration and travel, adds complexity. National-level heterogeneity highlights the need for tailored healthcare policies. Global efforts should prioritize prevention from birth, focusing on education to discourage raw pork consumption and raise awareness among children and young adults. For CIE patients, a tiered approach is recommended, combining antiepileptic drugs, anthelmintic treatment, and corticosteroids to manage seizures and reduce inflammation. Regular medication adherence and follow-up are essential for maintaining quality of life. Future directions include targeted and gene therapies. Trained social workers can enhance these efforts through community outreach, education, screening, and facilitating healthcare access for high-risk populations.

## Data Availability

The datasets presented in this study can be found in online repositories. The names of the repository/repositories and accession number(s) can be found below: vizhub.healthdata.org/gbd-results/.

## Footnotes

^1^ http://ghdx.healthdata.org/gbd-results-tool

^2^ https://www.healthdata.org/gbd/methods-appendices-2021/epilepsy

^3^ https://www.healthdata.org/gbd/methods-appendices-2021/cysticercosis-0

^4^ https://cran.r-project.org/web/packages/forecast/

^5^ https://cran.r-project.org/web/packages/tseries/

## Supplementary material

SUPPLEMENTARY FIGURE S1 The prevalence and YLDs numbers **(A,B)** and ASIR **(C,D)** of CIE in global and the five SDI regions from 1992 to 2021.

SUPPLEMENTARY FIGURE S2 Temporal trend of CIE burden in global. **(A)** Prevalence cases and rates from 1992 to 2021. **(B)** YLDs cases and rates from 1992 to 2021.

SUPPLEMENTARY FIGURE S3 Temporal trend of CIE burden in21 regions. **(A,B)** Prevalence cases and rates in 2021. **(C,D)** YLDs cases and rates in 2021.

SUPPLEMENTARY FIGURE S4 Number and ASR of prevalence **(A,B)** and YLDs **(C,D)** of CIE by age and sex in 2021.

SUPPLEMENTARY FIGURE S5 The associations between the SDI and ASR per 100,000 population of CIE across 204 GBD countries. (A) Prevalence; (B) YLDs. SDI = Socio-Demographic Index, GBD = Global Burden of Disease; YLDs = Years lived with disability.

SUPPLEMENTARY FIGURE S6 Age-period-cohort effects on CIE YLDs globally. **(A)** Age effects are shown by the fitted longitudinal age curves of YLDs (per 100,000 person-years) adjusted for period deviations. **(B)** Period effects are shown by the relative risk of YLDs (YLDs rate ratio) and computed as the ratio of age-specific rates from 1992 to 1996 (the referent period) to 2017–2021. **(C)** Cohort effects are shown by the relative risk of YLDs and computed as the ratio of age-specific rates from the 1895 cohort to the 2012 cohort.

SUPPLEMENTARY FIGURE S7 Prevalence and YLDs number of CIE in global and 5 SDI region from 1992 to 2036.

SUPPLEMENTARY FIGURE S8 Joinpoint regression analysis of the trends of ASR **(A,B)** and number **(C,D)** of prevalence and YLDs of CIE in global and 5 SDI region from 1992 to 2021.

## Glossary

YLDs: years lived with disability
CIE: cysticercosis-induced epilepsy
SDI: sociodemographic index
ARIMA: Auto Regressive Integrated Moving Average
ASR: age-standardized rates
GBD: Global Burden of Disease
NCC: neurocysticercosis
GHDx: Global Health Data Exchange
ASPR: age-standardized prevalence rate
ASYR: age-standardized YLDs rate
APC: annual percentage change
AAPC: average annual percentage change
MRI: magnetic resonance imaging
CT: computerized tomography; HALE, healthy life expectancy
IHME: the Institute for Health Metrics and Evaluation
DisMod-MR: Disease Modeling Meta-Regression
ICD-10: The International Classification of Diseases, Tenth Revision
MR-BRT: metaregression-Bayesian, regularized, trimmed
UIs: uncertainty intervals
EAPC: estimated annual percentage change
CIs: confidence intervals
ACF: autocorrelation function
PACF: partial autocorrelation function
AIC: Akaike information criterion
BIC: Bayesian Information Criterion
