# Supplementary figures and images for: Temporal trends and future projections of cysticercosis-induced epilepsy: insights from the global burden of disease study 2021- a cross-sectional study
Source: Front Public Health. 2025 May 19;13:1576226. doi: 10.3389/fpubh.2025.1576226 (PMC12127350; doi:10.3389/fpubh.2025.1576226)

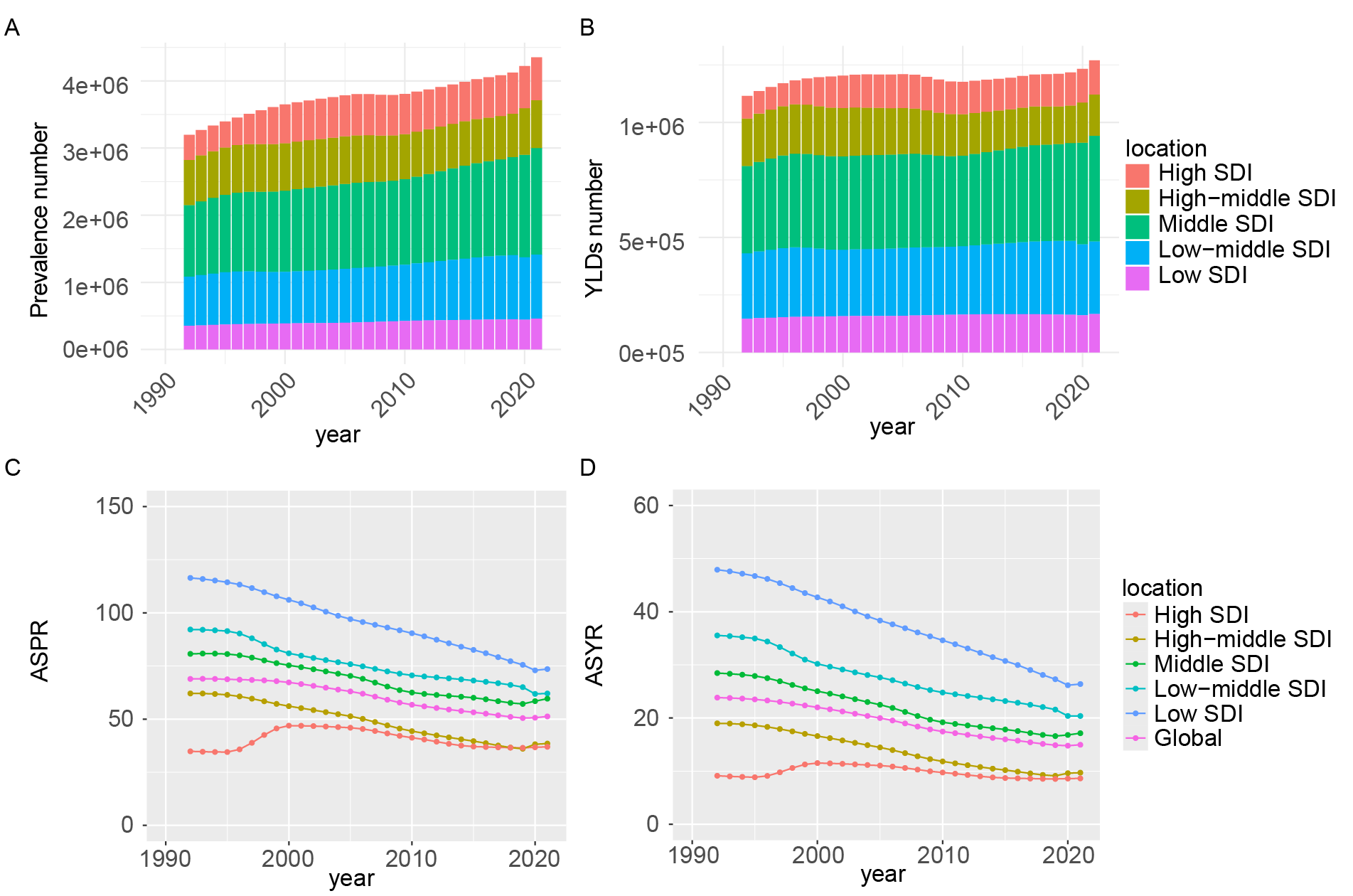

Supplement: SUPPLEMENTARY FIGURE S1 — The prevalence and YLDs numbers (A,B) and ASIR (C,D) of CIE in global and the five SDI regions from 1992 to 2021. [file Image_1.TIF]

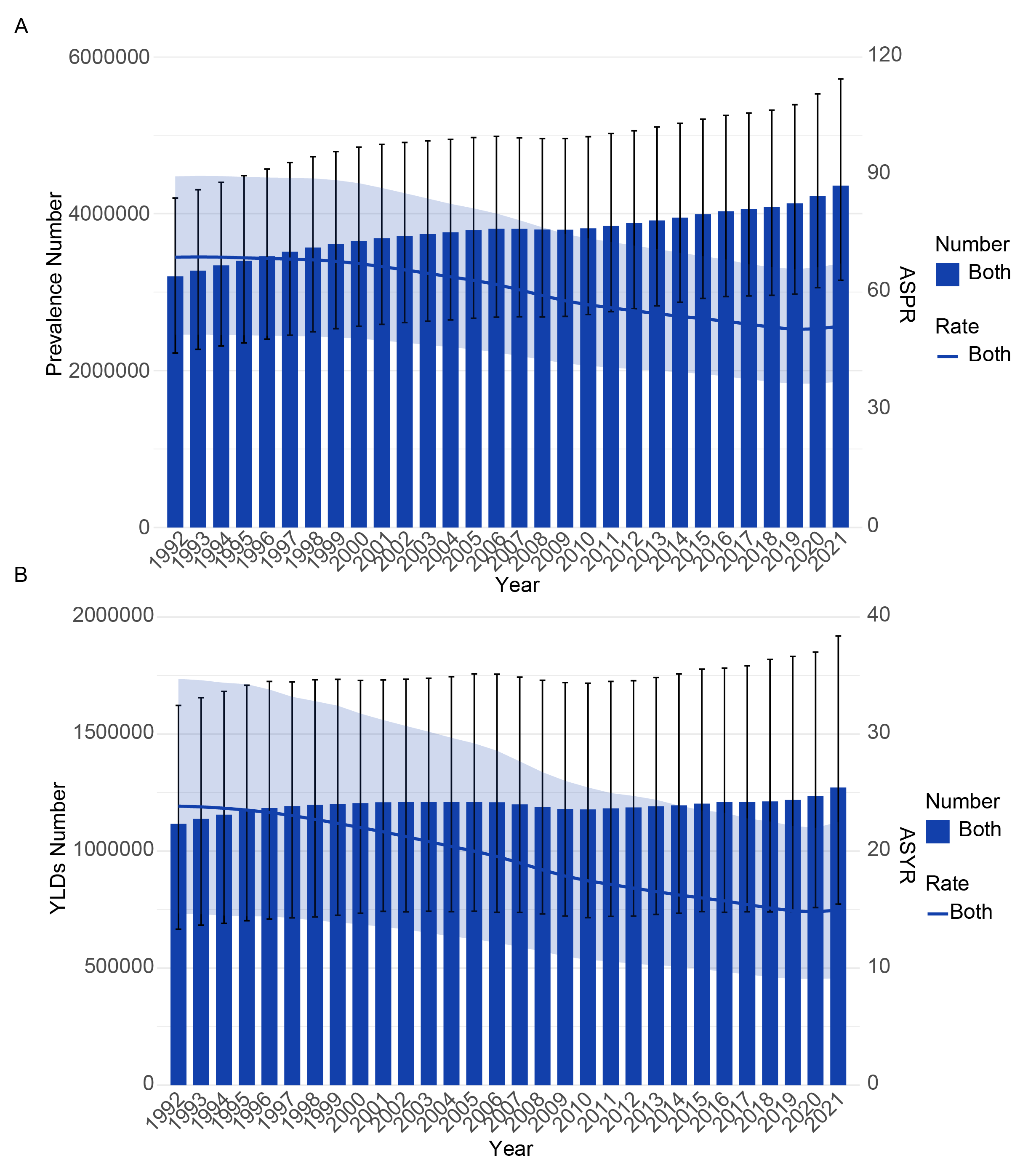

Supplement: SUPPLEMENTARY FIGURE S2 — Temporal trend of CIE burden in global. (A) Prevalence cases and rates from 1992 to 2021. (B) YLDs cases and rates from 1992 to 2021. [file Image_2.TIF]

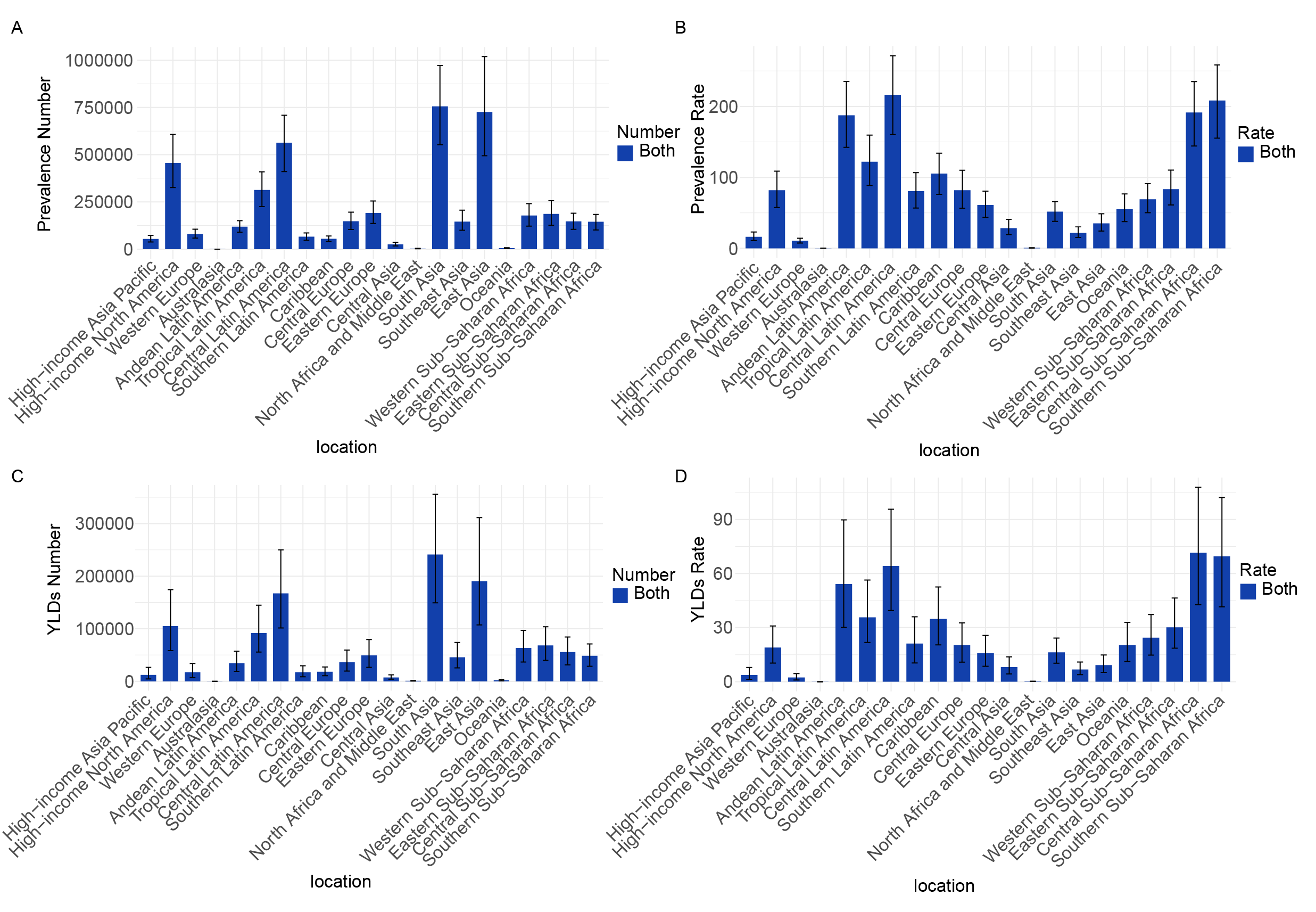

Supplement: SUPPLEMENTARY FIGURE S3 — Temporal trend of CIE burden in21 regions. (A,B) Prevalence cases and rates in 2021. (C,D) YLDs cases and rates in 2021. [file Image_3.TIF]

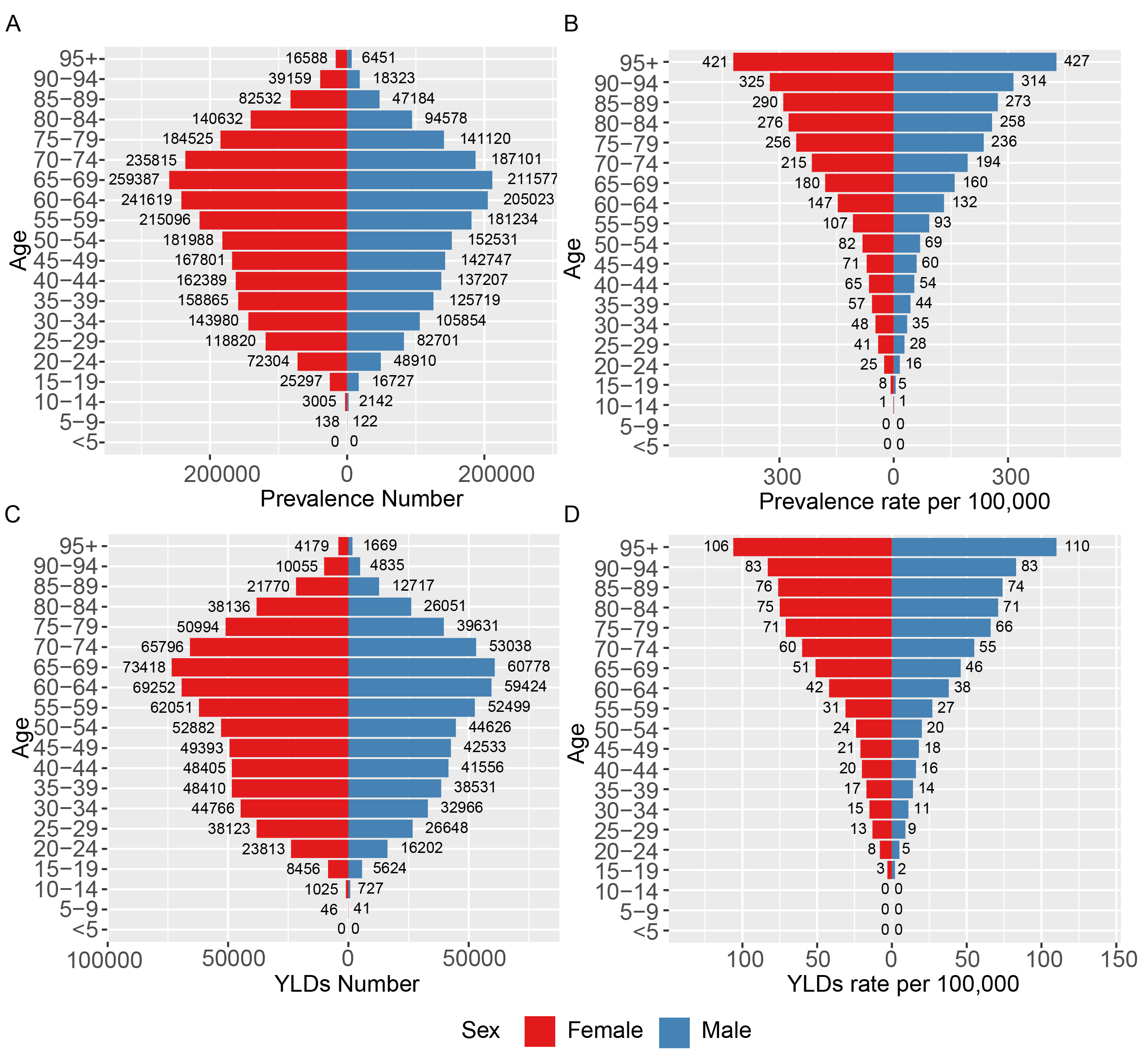

Supplement: SUPPLEMENTARY FIGURE S4 — Number and ASR of prevalence (A,B) and YLDs (C,D) of CIE by age and sex in 2021. [file Image_4.TIF]

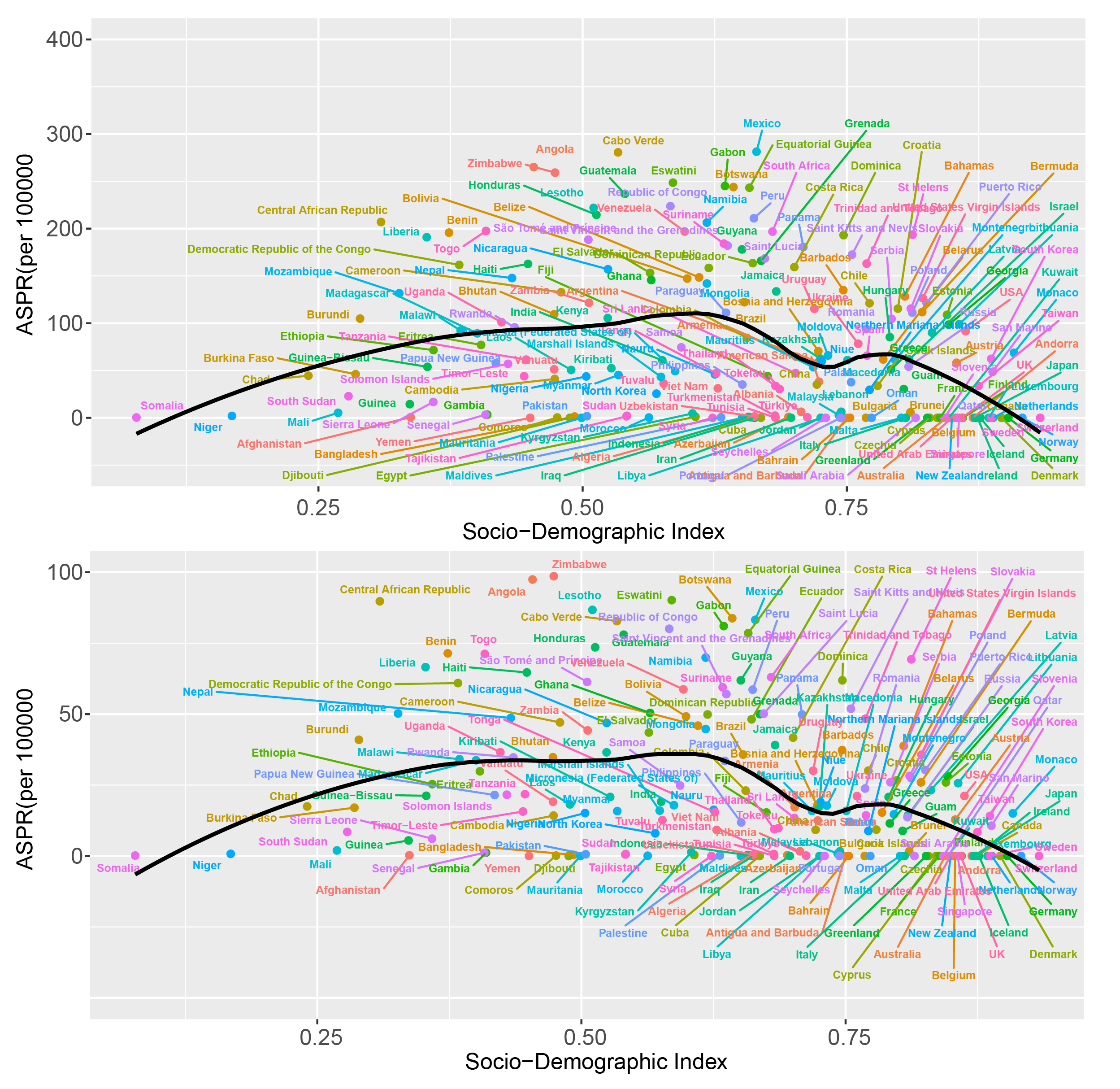

Supplement: SUPPLEMENTARY FIGURE S5 — The associations between the SDI and ASR per 100,000 population of CIE across 204 GBD countries. (A) Prevalence; (B) YLDs. SDI = Socio-Demographic Index, GBD = Global Burden of Disease; YLDs = Years lived with disability. [file Image_5.TIF]

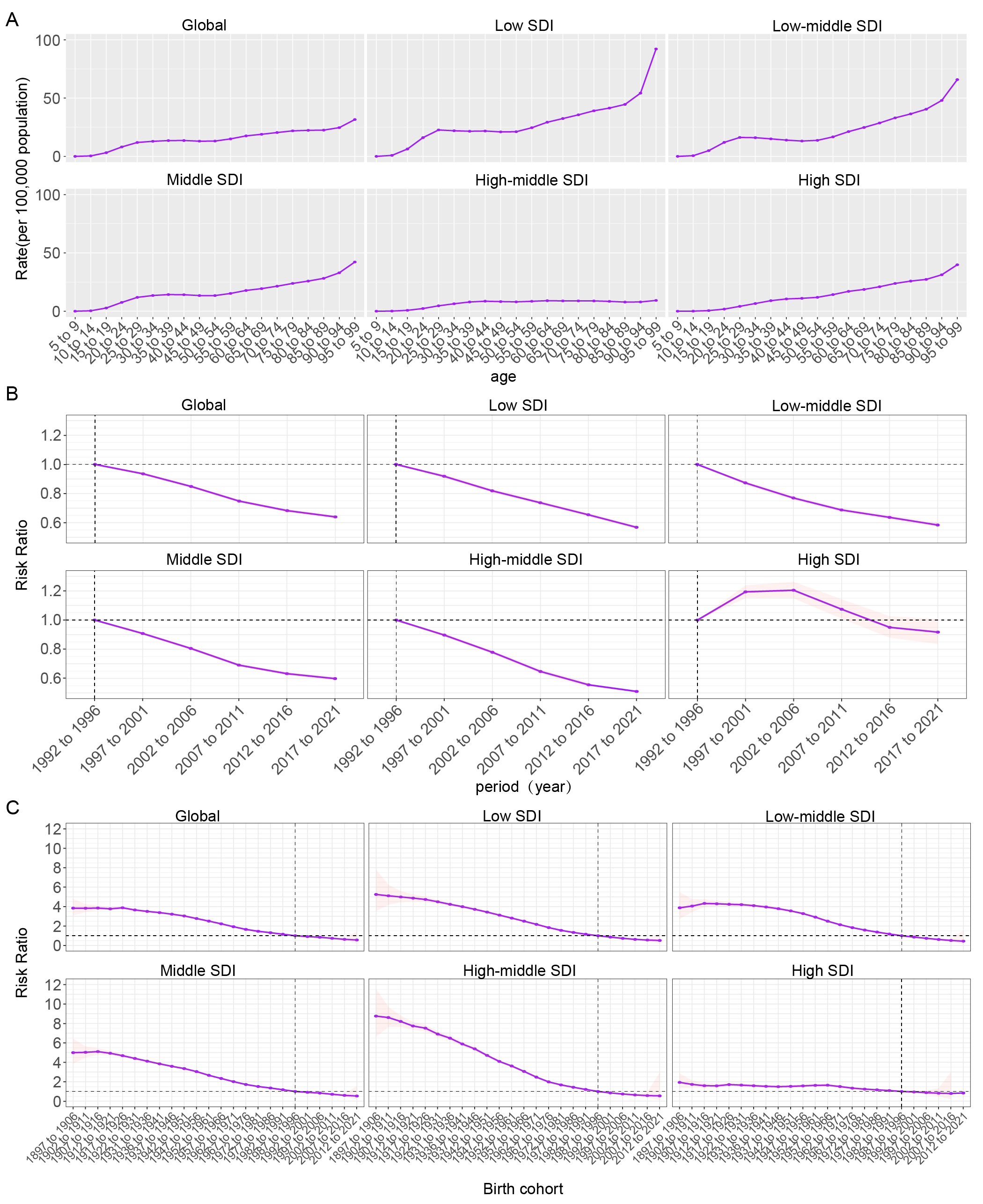

Supplement: SUPPLEMENTARY FIGURE S6 — Age-period-cohort effects on CIE YLDs globally. (A) Age effects are shown by the fitted longitudinal age curves of YLDs (per 100,000 person-years) adjusted for period deviations. (B) Period effects are shown by the relative risk of YLDs (YLDs rate ratio) and computed as the ratio of age-specific rates from 1992 to 1996 (the referent period) to 2017–2021. (C) Cohort effects are shown by the relative risk of YLDs and computed as the ratio of age-specific rates from the 1895 cohort to the 2012 cohort. [file Image_6.TIF]

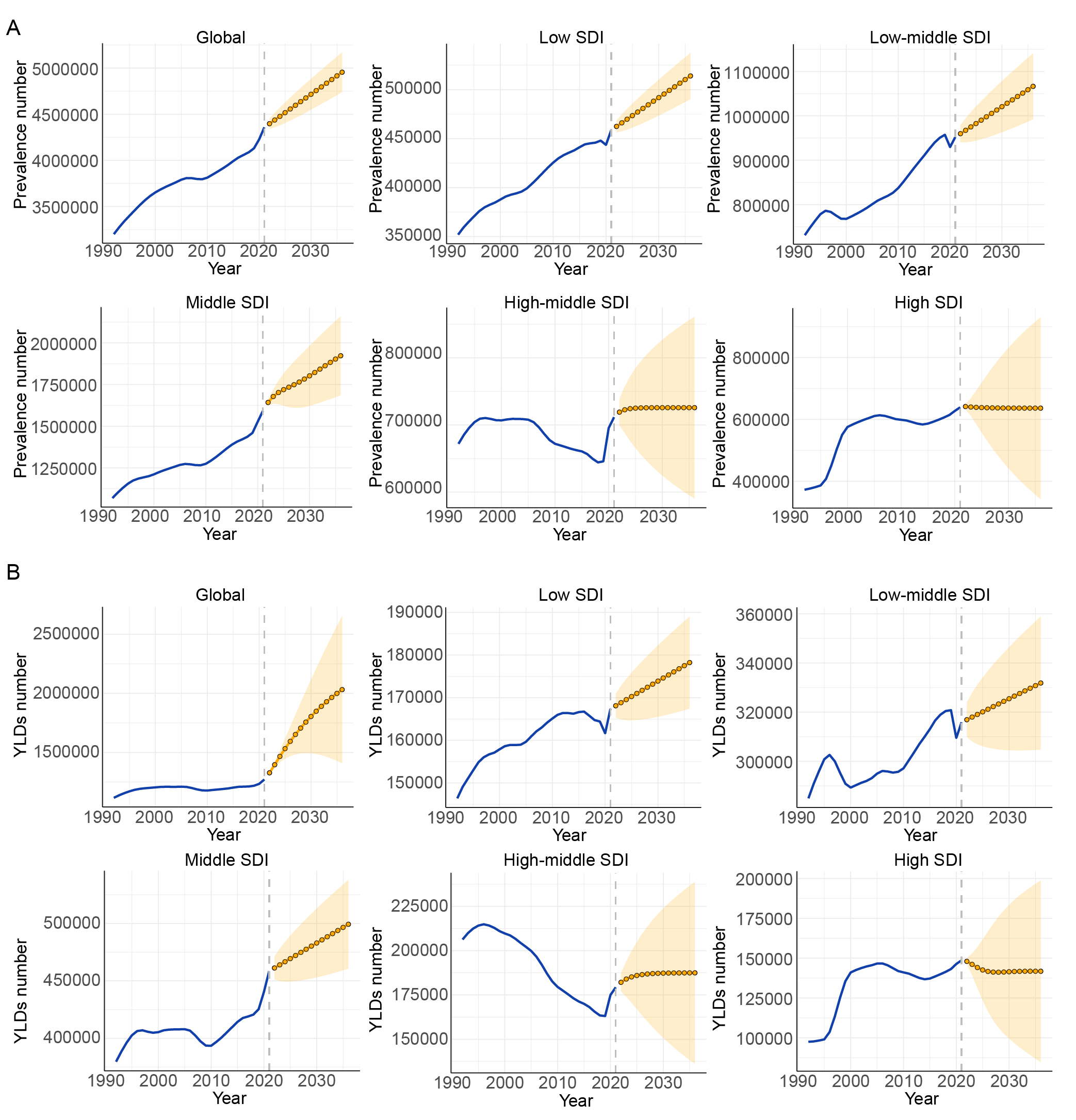

Supplement: SUPPLEMENTARY FIGURE S7 — Prevalence and YLDs number of CIE in global and 5 SDI region from 1992 to 2036. [file Image_7.TIF]

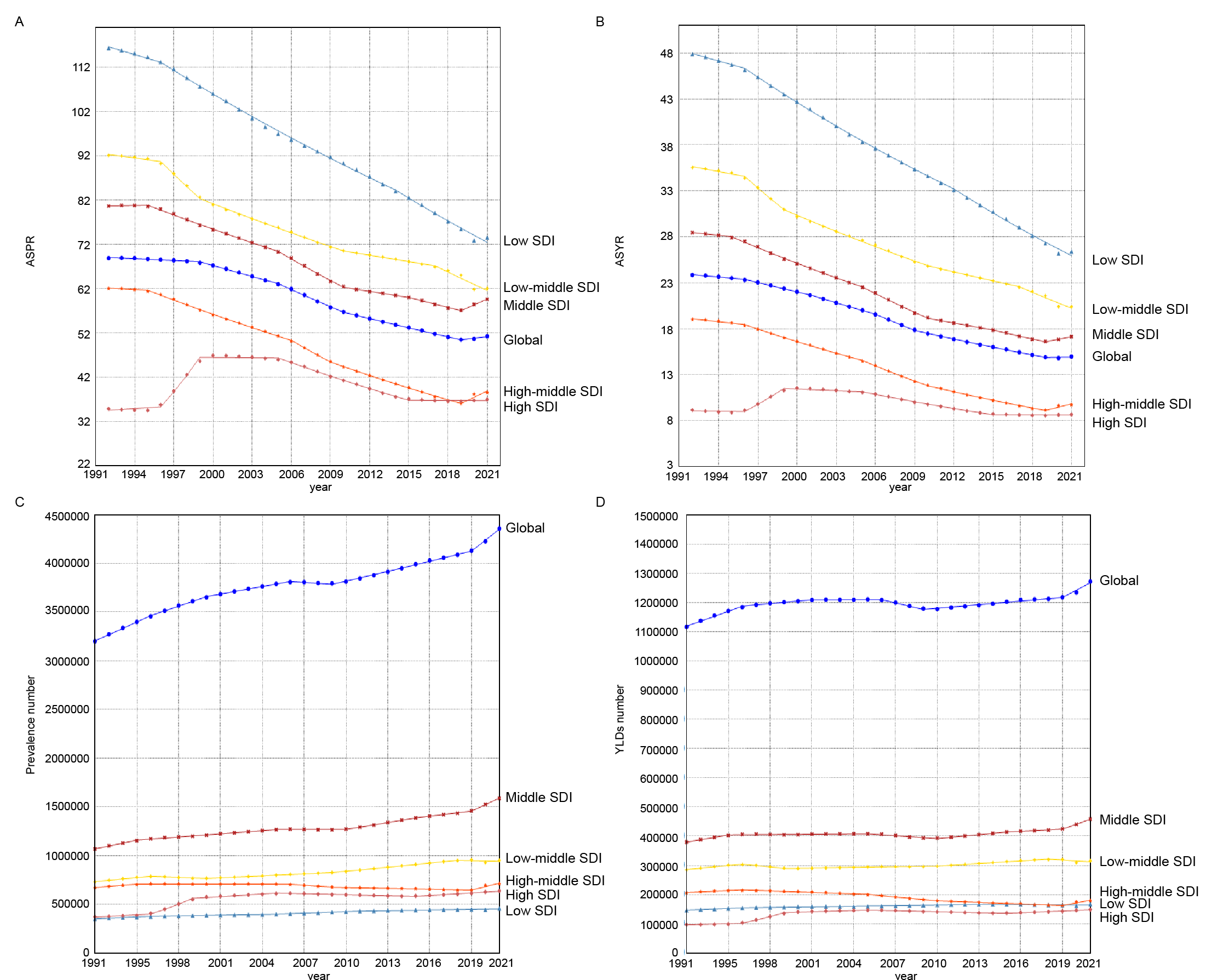

Supplement: SUPPLEMENTARY FIGURE S8 — Joinpoint regression analysis of the trends of ASR (A,B) and number (C,D) of prevalence and YLDs of CIE in global and 5 SDI region from 1992 to 2021. [file Image_8.TIF]
